# Discontinuation of Anticholinergic Medications Among VA Nursing Home Residents With and Without Dementia

**DOI:** 10.1093/geroni/igaf122.357

**Published:** 2025-12-31

**Authors:** Cellas Hayes

**Affiliations:** Stanford University, Stanford, California, United States

## Abstract

Anticholinesterase drugs are used commonly among older adults despite their known adverse effects on cognitive outcomes. A major challenge in studying their use is the difficulty of capturing over-the-counter anticholinergic medications, such as antihistamines, in electronic health records. In Veterans Affairs (VA) nursing homes, healthcare providers administer and track medications using a barcode medication administration system, which allows us to capture all anticholinergic medications, including over-the-counter ones. The present study used data from 45,183 VA long-term care residents (admitted between 2006 – 2019) aged 65 years and older. We captured antihistamine and non-antihistamine anticholinergic medication use, and linked with data on demographics, chronic conditions, laboratory measures, and functional status data from the minimum dataset. Residents were an average of 78.1 years old and 2.5% were women and 44.5% had dementia. The median length of stay was 9 months. Residents with dementia used antihistamine anticholinergics for a median of 6.5% (0.15–100%) weeks of their stay, compared to 10.7% (0.13–100%) in those without dementia. Additionally, 44% of residents with dementia used non-antihistamine anticholinergic medications at some point during their stay compared with 56% of those without dementia. Over 80% of residents with and without dementia discontinued their non-antihistamine anticholinergic medication before discharge or death. Anticholinergic medication use is high among nursing home residents but discontinuation is common.

